# Three new cases of late-onset cblC defect and review of the literature illustrating when to consider inborn errors of metabolism beyond infancy

**DOI:** 10.1186/s13023-014-0161-1

**Published:** 2014-11-15

**Authors:** Martina Huemer, Sabine Scholl-Bürgi, Karine Hadaya, Ilse Kern, Ronny Beer, Klaus Seppi, Brian Fowler, Matthias R Baumgartner, Daniela Karall

**Affiliations:** Division of Metabolic Diseases and Children’s Research Center, University Children’s Hospital Zürich, Zürich, Switzerland; Radiz – Rare Disease Initiative Zürich, University Zürich, Zürich, Switzerland; Department of Pediatrics, LKH Bregenz, Bregenz, Austria; Clinic for Pediatrics I; Inherited Metabolic Disorders, Innsbruck Medical University, Innsbruck, Austria; Divisions of Nephrology and Transplantation, Geneva University Hospitals, Geneva, Switzerland; Pediatric Nephrology & Metabolism, Children’s Hospital, University of Geneva, Geneva, Switzerland; Department of Neurology, Innsbruck Medical University, Innsbruck, Austria

**Keywords:** Vitamin B12, Cobalamin, Psychiatric disorder, Myelopathy, Funicular myelosis, Haemolytic uraemic syndrome, Thrombosis, Pulmonary artery hypertension

## Abstract

**Background:**

The cblC defect is a rare inborn error of intracellular cobalamin metabolism. Biochemical hallmarks are elevated homocysteine and low methionine in plasma accompanied by methylmalonic aciduria. Due to the heterogeneous clinical picture, patients with the late-onset form of the disease (onset >12 months) come to the attention of diverse medical specialists, e.g. paediatricians, neurologists, nephrologists, psychiatrists or haematologists. The report reviews the published clinical data and adds three new cases to raise awareness for this severe but often treatable disease.

**Methods:**

The Pubmed and the Cochrane databases were searched for clinical reports on cblC patients and three unreported cases are presented to illustrate the clinical spectrum.

**Results:**

Reports on 58 cases (30 females, 22 males, 6 = no information) and the three new cases underlined the clinical heterogeneity of the disease. Time between first symptoms and diagnosis ranged from three months to more than 20 years. Haemolytic uraemic syndrome and pulmonary hypertension were main presenting symptoms in preschool children. In older children/adolescents, psychiatric symptoms, cognitive impairment, ataxia and myelopathy were frequently observed while thromboembolic events and glomerulopathies were almost exclusively seen in adults. Brain atrophy, white matter lesions and myelopathy were frequently encountered. The majority of patients showed marked biochemical and clinical response to treatment with parenteral hydroxocobalamin combined with oral betaine, folate, carnitine and rarely methionine. The course was less favourable in late treated or untreated patients.

**Conclusions:**

The late-onset cblC defect is a rare disease and unfortunately, diagnosis is often delayed. Raising awareness for this disorder can significantly improve patients’ outcome and perspective by timely initiation of targeted treatment. Newborn screening (NBS) for the cblC defect might be of benefit especially for late-onset patients since treatment seems efficient when initiated before irreversible organ damage. In general, inborn errors of metabolisms should be considered in unexplained medical cases at any age, especially in patients with multisystemic disease. More specifically, total homocysteine in plasma and methylmalonic acid in urine/plasma should be measured in unexplained neurologic, psychiatric, renal, haematologic and thromboembolic disease.

## Background

The cblC defect (ORPHA 79282; MIM 277400) is an inborn error of intracellular cobalamin (cbl) metabolism caused by mutations in the *MMACHC* gene, which is located on chromosome 1p34.1 [1]. Due to the defective *MMACHC* gene product, methylcobalamin and adenosylcobalamin cannot be synthesized. Since methylcobalamin is the essential cofactor for the enzyme methionine synthase, the defect causes an impairment of the remethylation of homocysteine (Hcy) to methionine (Met). Adenosylcobalamin is cofactor for the mitochondrial enzyme methylmalonyl-CoA mutase, a critical enzyme in the degradation of methylmalonic acid (MMA) [2]. Biochemical markers for the disease are elevated total Hcy (tHcy) in plasma and MMA in plasma and urine in the presence of low plasma Met concentrations.

Approximately 90% of reported patients with the cblC defect present with the severe, infantile, early-onset form of the disease [3,4]. Characteristic clinical manifestations of the infantile onset cblC defect are feeding difficulties and failure to thrive, developmental delay, microcephaly, seizures and muscular hypotonia, decreased visual acuity due to pigmentary retinopathy and nystagmus. The true incidence of the late-onset cblC defect is probably higher than presently reported since in many cases the diagnosis may be missed.

The early-onset form of the cblC defect is associated with the frameshift mutation c.271dupA in Europeans, with the c.331C > T nonsense mutation in the French-Canadian, Acadian and Cajun populations, and with the c.609G > A nonsense mutation in the Han Chinese population. The c.394C > T nonsense mutation is encountered most frequently in late-onset patients from Portugal, Italy, Arabian and Asian countries, while the c.482G > A missense mutation has been reported in single patients from Europe [5,6] and China [7]. There is evidence that the c.394C > T mutation might result in a truncated protein with residual function thus explaining its association with late rather than early onset disease. *MMACHC* mRNA expression studies showed that cell lines harbouring the c.394C > T mutation had significantly higher levels of transcript compared to cells carrying mutations associated with early-onset disease [6]. In patients who are compound heterozygous for e.g. a missense mutation and c.271dupA, the phenotype seems to be predominantly determined by the “milder” mutation [5].

In two large series of cblC patients reported in the literature, 26% [3] and 11.4% [4] of the patients – the great majority with disease onset in the first year of life - died. In survivors, neurological and eye disease progressed despite treatment [4]. Even in infants diagnosed by newborn screening and treated early in life, neurological and eye disease progresses and long-term neurocognitive outcome is significantly impaired [8]. In the above cited series, onset was classified as late in six out of 60 patients [3], with first symptoms between age four and fourteen and in ten out of 88 patients [4] with onset between > twelve months and 13.5 years respectively. Other late-onset cases have been presented as case reports or small series. Clinical manifestations in late-onset patients are significantly different from the early-onset form and very heterogeneous [4] and thus late-onset disease may be underdiagnosed.

The aims of this report are to i) systematically review published data on the clinical spectrum of the late-onset cblC defect; ii) evaluate treatment approaches, biochemical and clinical response and general outcome; iii) illustrate the types of clinical presentation; iv) discuss ways to diagnosis and highlight clinical signs which should prompt the consideration of inborn errors of metabolism (IEM) beyond infancy.

## Methods

The Pubmed and the Cochrane databases were searched using the terms “cobalamin; cblC; *MMACHC*, combined methylmalonic aciduria homocystinuria, MMA, homocysteine”. The terms were combined with “adult onset, late onset, review, case, case series”. In addition, references listed in the papers retrieved by this method were screened. All cases with onset of the disease >12 months were included [4]. Data on clinical symptoms and biochemical and genetic data were pooled for analysis whenever the description of the cases allowed.

Written informed consent was obtained from all three patients for publication of their case reports.

## Results

Twenty-five reports on cases and case series were selected as relevant to the topic and containing details on clinical signs and symptoms in late-onset cblC cases. Data on all parameters of interest where not consistently given in the reports and are thus reported for varying numbers of cases according to availability of data.

The reports covered 58 cases, 30 females and 22 males [no information on gender available for six patients]. Three clinically asymptomatic individuals but with the typical biochemical profile of the disease were reported [9-11]. One clinically asymptomatic female had been identified by investigations following the observation of low free carnitine in her child’s newborn screening [11]. One female and one male asymptomatic patient respectively were diagnosed by family screening after diagnostic workup in their symptomatic siblings [9,10].

The male (n = 21) to female (n = 28) distribution in 49 symptomatic patients was uneven (ratio 0.75).

**Figure 1:**
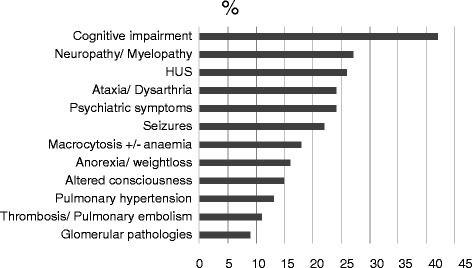
**Frequency of clinical signs and symptoms in 55 patients with the late-onset cblC defect.**

Table 1 summarizes the information on gender, age and clinical signs at presentation, treatment and outcome in the published cases (not including the three new cases presented in this report). Descriptions of clinical signs and symptoms were extracted from reports on 55 symptomatic patients [Figure 1]. Overall, cognitive decline/impairment was the most frequent symptom, followed by neurological symptoms such as affections of the spinal cord myelin, ataxia and seizures. Haemolytic uraemic syndrome (HUS) and pulmonary arterial hypertension (PAH) were often associated. Macrocytosis or macrocytic anaemia were present in less than 20% of cases. Glomerulopathies as well as thromboembolic events were rarely observed.

**Table 1:** **Age at first symptoms, clinical presentation, treatment and outcome in 58 late-onset cblC cases**

**No.**	**Reference**	**Age at onset**	**Sex**	**Clinical presentation**	**Treatment**					**Outcome**
		**Years**			**OH-Cbl**	**Betaine**	**Folate**	**Carnitine**	**Met**	
1	[12]	1.25	F	PAH	x					Complete recovery
2	[13]	1.5	M	PAH, HUS	Untreated					Deceased without diagnosis
3	[7]	1.5	F	No information available	No information available					No information available
4	[7]	1.5	F	No information available	No information available					No information available
5	[7]	2	M	Lethargy, convulsions, hypotonia	No information available					No information available
6	[7]	2	M	No information available	No information available					No information available
7	[10]	2,5	M	Asymptomatic	Untreated					No information available
8	[13]	2,5	M	PAH, HUS	x					Deceased
9	[14]	3	M	PAH, HUS	Untreated					Deceased without diagnosis
10	[13]	3	M	PAH, HUS	Untreated					Deceased without diagnosis
11	[7]	3.5	M	Cognitive decline, lethargy, convulsions	No information available					No information available
12	[13]	4	F	PAH, HUS	x					Progressive PAH
13	[15]	4	F	HUS	x	x	x			Complete recovery
14	[16]	4	F	Cognitive decline, neuropathy, ataxia	x	x	x			Improvement, mild cognitive impairment, neurological sequelae
15	[17]	6	F	HUS	x	x	x			Chronic renal failure
16	[18]	7	F	Neuropathy, myelopathy, cognitive impairment, epilepsy	x		x			Cognition improved, seizures resolved, neurological sequelae
17	[17]	8	F	HUS	x	x	x			Renal parameters improved
18	[19]	10	F	Acute cognitive decline, anorexia, catatonia, psychosis, seizures. Brain volume loss, thinned corpus callosum	x	x				Seizures and psychiatric symptoms improved
19	[20]	11	F	Cognitive decline, behavioral changes, ataxia, myoclonic jerks	x	x	x	x		Complete recovery
20	[15]	11	M	HUS, hypertensive encephalopathy, coma, convulsions	x	x	x			Complete recovery (but antihypertensive drugs necessary)
21	[9]	12	F	Ataxia, neuropathy, myelopathy, mild neuropsychiatric symptoms.	x					Improved; neurological sequelae
22	[21]	13	F	Cognitive decline, ataxia/dysarthria, EEG abnormal	x					Cognition improved, neurological sequelae
23	[13]	14	F	PAH, HUS	x					“Stable” disease
24	[19]	14	F	Cognitive decline, depression, ataxia, seizures, neuropathy/myelopathy	x	x		x		Improved; neurological sequelae
25	[10]	14	M	Acute psychosis, mental retardation	Untreated					No information available
26	[22]	16	F	Thromboembolism, neuropathy, myelopathy, psychiatric symptoms	x	x	x	x		Disease progression
27	[23]	16	M	Atypical glomerulopathy	x	x			x	Coma, deceased
28	[24]	18	F	Glomerulonephritis; psychiatric symptoms, cognitive impairment, recurrent thromboses, pulmonary embolism, seizures, neuropathy, myelopathy, cortical atrophy, leukoencephalopathy, corpus callosum agenesis	x	x	x			Deceased after initial improvement
29	[25]	20	M	HUS, renal failure, malignant hypertension	x	x	x			Improved, renal function stable
30	[26]	20	M	Neuropathy, myelopathy, progressive encephalopathy, confusion, deep venous thrombosis, progressive respiratory failure	x			x		Improved, neurological sequelae
31	[18]	22	F	Triggered by pregnancy/caesarian section: Sluggish response, neuropathy	x		x			Complete recovery
32	[27]	23	M	Cognitive impairment, ataxia, neuropathy, spinal cord myelin lesions	x	x	x	x		Improved, neurological sequelae
33	[22]	24	F	Myelopathy	x	x	x	x		Moderate myelopathy
34	[9]	29	F	Asymptomatic	Untreated					Asymptomatic
35	[11]	29	F	Asymptomatic	x					Biochemical response
36	[28]	32	F	Progressive neuropathy, myelopathy, optic disk pallor, leukopenia	Untreated					Deceased without diagnosis
37	[24]	33	F	Glomerulonephritis, recurrent deep venous thrombosis.	x	x	x			No more thromboses
38	[29]	36	F	Neuropathy, psychiatric symptoms	x	x	x			Mental status improved, neurological sequelae
39	[30]	38	M	Hypertension, seizures, progressive confusion, progressive periventricular white matter lesions	x	x	x			Complete recovery
40	[18]	40	M	Cognitive decline, hallucinations, neuropathy, myelopathy, brain atrophy.	x		x			Complete recovery
41	[24]	41	M	Depression, neuropathy, myelopathy, periventricular leucoencephalopathy, abnormal signal in spinal cord myelin	x	x	x			Overall improvement. Spinal cord myelin lesion disappeared
42	[28]	44	F	Cognitive decline, optic disk pallor, venous thrombosis, pulmonary embolism	Untreated					Deceased without diagnosis
43-48	[3]	4-14	n.a.	No individual information reported	No information available					N = 5 “very positive”; n = 1 “moderately impaired”
49-58	[4]	1-13.5	5 M	No individual information reported	No information available					Overall reduction of symptoms
			5 F

**Figure 2:**
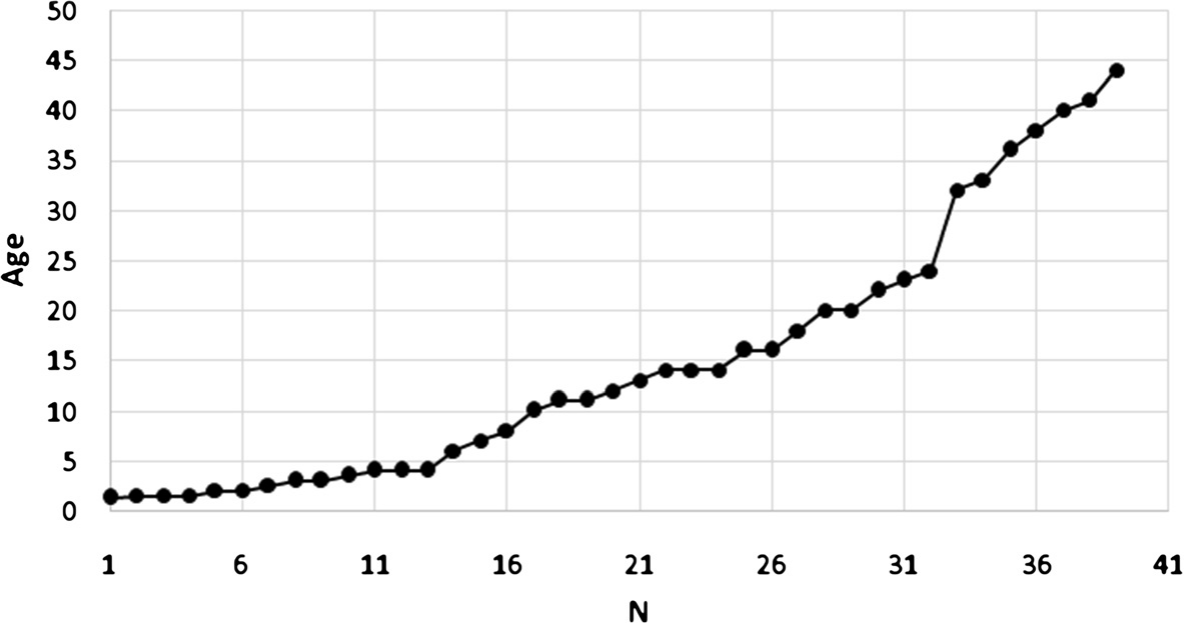
**Age at disease onset (cumulative) in 39 patients with the late-onset cblC defect.**

**Figure 3:**
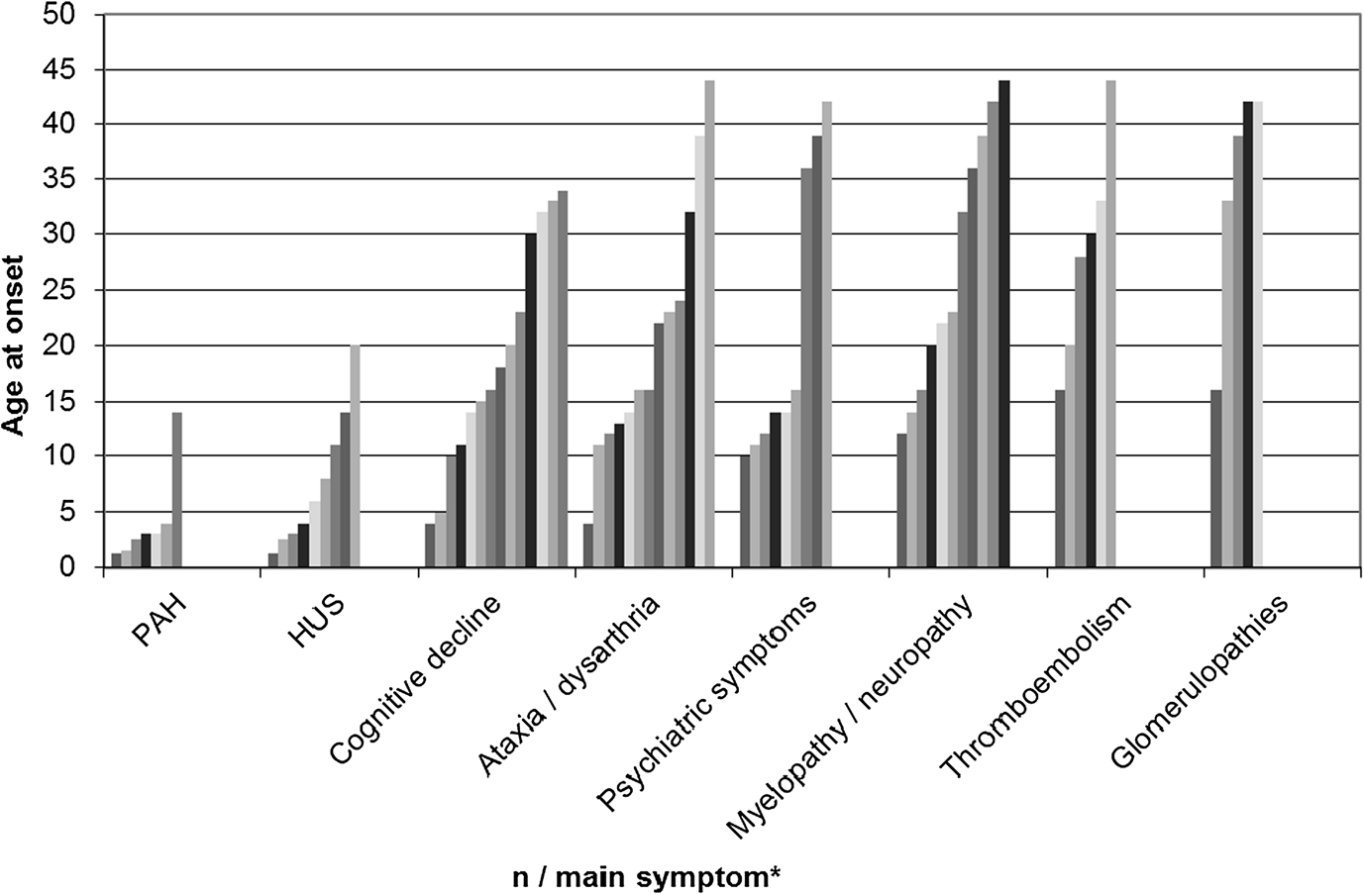
**Number of patients and age at onset for main clinical symptoms in 39 patients with the late-onset cblC defect.** * Each column represents one patient.

Information on age at disease onset was available for 39 symptomatic patients (mean 15 years; median 12 years, range 1.3-44 years) [Figure 2]. The frequency of symptoms differs markedly over the lifespan. Age specific patterns of the disease are depicted in Figure 3. HUS (median age at onset 6; mean 7.8, range 1.25-20 yrs) and PAH (median age at onset 3; mean 4, range 1.25-14 yrs) were the most frequent symptoms in very young children. In older children/adolescents psychiatric symptoms (median age at onset 14; mean 21.6, range 10-42 yrs), ataxia/dysarthria (median age at onset 16; mean 20.8, range 4-44 yrs) and cognitive decline (median age at onset 17; mean 19, range 4-34 yrs) were most frequent; while in adults, in addition to cognitive decline and ataxia/dysarthria, thromboembolic events (median age at onset 29; mean 28.5, range 16-44 yrs), neuropathy/myelopathy (median age at onset 23; mean 27.3, range 12-44 yrs) and non-HUS renal disease (glomerulopathies) (median age at onset 39; mean 34.4, range 16-42 yrs) were dominant features.

Outcome of the disease ranged from death in eight patients (in five of them, the diagnosis could only be established post mortally) to complete recovery in seven treated patients. In the reviewed sample, neurological and cognitive improvement occurred in eleven patients; renal functions stabilized in two patients. Clinical symptoms were considered to generally respond to treatment in the cohorts reported by Fischer et al. [4] and Rosenblatt et al. [3] [Table 1].

Magnetic resonance imaging (MRI) findings were reported in more detail for seventeen patients. Normal brain imaging was present in two symptomatic patients. Brain volume loss was observed in eight patients. Brain white matter lesions were present in seven patients and periventricular (nearly) confluent white matter hyperintensities in T2 images seemed a distinct pattern. Spinal cord myelin pathology was reported in four cases, labelled as spinal infarct in one and described as high intensity signals in T2-weighted images in three patients.

Time between first symptoms and diagnosis was mentioned in the reports on eighteen cases and ranged from three months to more than 20 years (data not shown).

At diagnosis, mean tHcy was 138 μmol/L (n = 31; range 28–309 μmol/L; *reference range* 5–15) and decreased in all patients under treatment to a mean of 35 μmol/L (n = 15; range 8–169 μmol/L). Mean Met was initially low with 11.5 μmol/L (n = 17; range 4–34 μmol/L; *reference range 20–45*) and increased to 33 μmol/L (n = 11; range 12.7-62 μmol/L). Mean MMA in urine at disease onset was 1950 μmol/mmol creatinine (n = 13; range 118–5558 μmol/mmol creatinine; *reference range < 10)* and decreased to 98 μmol/mmol creatinine during follow-up (n = 7; range 5.8-250 μmol/mmol creatinine).

Information on genotype was available for 42 individuals, including the three new cases presented in this paper. The c.271dupA (21%), c.394C > T (21%) and c.609G > A (11%) were the most frequent mutated alleles in this cohort of late-onset patients. Homozygosity for the c.394C > T mutation –a genotype which has been described before to be associated with the late-onset cblC defect [5]- was present in seven individuals and thus constituted the most frequent genotype in this population. Homozygosity for the c.271dupA mutation, which is associated with the early-onset cblC defect, was present only in a single late-onset patient [Table 2].

**Table 2:** **Genotypes of 42 patients with the late onset cblC defect**

**Allele 1**			**Allele 2**			**N**	**Reference**
**Nucleotide change**	**Amino acid change**	**Exon**	**Nucleotide change**	**Amino acid change**	**Exon**		
c.271dupA	p.Arg91Lysfs*14	2	c.271dupA	p.Arg91Lysfs*14	2	1	[4]
c.271dupA	p.Arg91Lysfs*14	2	c.276G > T	p.Glu92Asp	2	2	[13]
c.271dupA	p.Arg91Lysfs*14	2	c.82-9_12delTTTC	r.spl?	Intron 1	1	[4]
c.271dupA	p.Arg91Lysfs*14	2	c.347 T > C	p.Leu116Pro	3	3	[4,22]
c.271dupA	p.Arg91Lysfs*14	2	c.365A > G	p.His122Arg	3	1	[24]
c.271dupA	p.Arg91Lysfs*14	2	c.389A > G	p.Tyr130Cys	3	1	[25]
c.271dupA	p.Arg91Lysfs*14	2	c.394C > T	p.Arg132*	3	1	[4]
c.271dupA	p.Arg91Lysfs*14	2	c.440G > C	p.Gly147Ala	4	2	[28]
c.271dupA	p.Arg91Lysfs*14	2	c.482G > A	p.Arg161Gln	4	2	[26,29]
c.271dupA	p.Arg91Lysfs*14	2	c.565C > A	p.Arg189Ser	4	1	[24]
c.271dupA/c.565C > A	p.Arg91Lysfs*14/	2	c.565C > A	p.Arg189Ser	4	1	[4]
c.276G > A	p.Glu926Glu [p.(=)]	2	c.442_444delinsA	p.Val148Metfs*33	4	1	[13]
c.276G > A	p.Glu926Glu [p.(=)]	2	c.14_24del11	p.Val5Glufs*25	1	1	[13]
c.276G > T	p.Glu92Asp	2	c.276G > T	p.Glu92Asp	2	1^#^	-
c.276G > T	p.Glu92Asp	2	c.442_444delinsA	p.Val148Metfs*33	4	1	[14]
c.347 T > C	p.Leu116Pro	3	c.347 T > C	p.Leu116Pro	3	1^#^	
c.365A > G	p.His122Arg	3	c.457C > T	p.Arg153*	4	1	[24]
c.392_394del	p.Gln131del	3	c.392_394del	p.Gln131del	3	1	[27]
c.394C > T	p.Arg132*	3	c.394C > T	p.Arg132*	3	7	[4,16,19]
c.394C > T	p.Arg132*	3	c.468_469delCT	p.Trp157Valfs*24	4	1	[4]
c.464G > A	p.Gly155Glu	4	c.464G > A	p.Gly155Glu	4	1	[13]
c.482G > A	p.Arg161Gln	4	c.82-1G > A	r.spl?	Intron 1	1^#^	-
c.482G > A	p.Arg161Gln	4	c.609G > A	p.Trp203*	4	2	[18]
c.484G > T	p.Gly162Trp	4	c.484G > T	p.Gly162Trp	4	1	[12]
c.609G > A	p.Trp203*	4	c.609G > A	p.Trp203*	4	2	[7]
c.609G > A	p.Trp203*	4	c.394C > T	p.Arg132*	3	1	[7]
c.609G > A	p.Trp203*	4	c.658_660delAAG	p.Lys220del	4	1	[7]
c.609G > A	p.Trp203*	4	c.1A > G	p.Met1?	1	1	[18]
c.1A > G	p.Met1?	1	n.d.			1	[7]

^#^new reported cases; n.d. = not detected.

### Illustrative unreported cases

#### Case 1: Psychiatric symptoms in adolescence and HUS in adulthood

A 23-year-old Portuguese male from a non-consanguineous family came to medical attention at the age of 23 years. History revealed that the patient’s sister had died at age 18 months from an unknown condition. The patient had had trouble at school due to attention deficits and aggressive behavior and left school at age fourteen. A depressive episode and excessive drug consumption occurred at age twenty. At age twenty-two, he was admitted to a psychiatric ward due to “strange” behavior, anxiety and signs of a depersonalization disorder.

On admission, the patient reported one-month history of progressive asthenia and shortness of breath when walking, upper abdominal pain, reduced appetite and unintended weight loss of 14 kg within two months. Arterial hypertension (blood pressure 200/110 mmHg) was present and ophthalmoscopy showed a grade III retinopathy with hemorrhages compatible with chronic hypertension. The patient had no impairment of visual acuity or nystagmus at any time.

Laboratory data on admission revealed macrocytic anemia (haemoglobin 73 g/L, mean corpuscular volume 99.5 fl), high level of reticulocytes (50.4%o, *reference range 5–15*), abundant fragmentocytes, and low level of platelets (88’000/mm^3^). Urea (21.7 mmol/L, *reference range 2.8-7.1)*, and creatinine (523 μmol/L, *reference range* 62*–*106), were significantly elevated and protein (56 g/L *reference range* 61–79), was low in the presence of proteinuria and haemoglobinuria. Renal and urinary tract ultrasound was unremarkable. Echocardiography showed marked concentric left ventricular hypertrophy with a moderately dilated left atrium. A total tHcy value of >250 μmol/L (*reference range* 5–15) was apparently unnoticed.

Under the diagnosis of malignant hypertension with secondary renal failure and/or HUS, antihypertensive therapy was initiated. Twenty-four hours after admission, the electrocardiogram showed inverted T waves in the presence of increased troponin levels and the patient was transferred to the intensive care unit. Blood pressure was difficult to stabilize even with consecutive and combined used of six antihypertensive drugs. At this time, the patient had two grand mal seizures.

End-stage renal failure developed within 15 days and renal replacement therapy was started. After six weeks in hospital, the patient was discharged with the diagnosis of malignant hypertension with secondary microangiopathy, renal failure, retinopathy and cardiomyopathy*.*

Fifteen months later, while clinically stable on renal replacement therapy, tHcy was measured as part of routine follow-up and the concentration was again highly elevated with 353 μmol/L. Met (16 μmol/L) was low and MMA was not assessed. The cblC defect was proven in cultured fibroblasts by Met and serine formation, [57Co]-Cobalamin uptake and coenzyme synthesis, and propionate incorporation [31]. Molecular genetic analysis revealed homozygosity for a c.565C > A (p.R189S) missense mutation in exon 4. This mutation had previously been described in compound heterozygosity in two Spanish late-onset patients [32], one late onset patient of unspecified origin [1] and two Portuguese early-onset patients [33].

Initially, tHcy levels around 37–66 μmol/L were achieved with daily IV OH-Cbl injections and oral betaine (16 g/d), carnitine (1 g/d) and folate (5 mg/d). Over the next five years, median tHcy level was 70 μmol/L (range24 to 149 μmol/L), median blood MMA was 18 μmol/L (ref <0.28) and median Met was 28 μmol/L under sometimes less than optimal compliance. In the meantime, the patient underwent renal transplantation and during the following five years, renal function was stable. Cardiac morphology normalized but antihypertensive treatment with a combination of three drugs remained required. Interestingly, psychosocial adaptation and functioning improved markedly. The patient became able to sustain a lasting relationship and family life, to keep social contacts and to pursue a successful professional career.

#### Case 2: Neuropathy, subacute and combined degeneration of the spinal cord, cognitive impairment, depression and thromboembolism

A previously healthy, twenty-six year old Austrian male presented with the clinical symptoms of a symmetric sensorimotor neuropathy. Family history was uneventful and without evidence for consanguinity. The condition was progressive and the diagnosis of an immune-mediated neuropathy was established and immunosuppressive treatment initiated. Nevertheless, the condition worsened and a myelopathy on basis of vitamin B12 deficiency was suspected, especially when the patient developed subacute thoracal myelopathy with hypoaesthesia, disturbance of position sense, pathological reflex pattern and sudden gait difficulties at the age of 30 years. The MRI scan of the myelon supported this idea but due to repeatedly normal vitamin B12 serum levels, the hypothesis was no longer pursued. At this time, the patient additionally complained about an unintended weight loss of 8 kg due to loss of appetite; impaired short-term memory, concentration problems and confusion. In parallel, the patient developed deep vein thrombosis, promoted by reduced physical activity following an ankle joint injury. Further investigations revealed peripheral pulmonary embolism, attributed to a heterozygous mutation in the *prothrombin* gene. Several months later due to progressive neurological and psychiatric symptoms, the patient was transferred for a second opinion to a tertiary center. Besides the sensory ataxic gait disorder due to neuropathy and myelopathy, the patient presented with depression and cognitive impairment including slow mentation, memory impairment, attention deficits, and executive impairment. Metabolic investigations considerably increased tHcy (228–264 μmol/L) low Met (7.7 μmol/L) and highly elevated urinary MMA excretion in qualitative analysis in the presence of normal vitamin B12 and holotranscobolamin levels. The cblC defect was proven by enzymatic and complementation studies in cultured fibroblasts as described [31]. Molecular genetic analysis of the *MMACHC* gene revealed a splice site mutation in intron 1 (c.82-1G > A) and a missense mutation in exon 4 (c.482G > A).

Treatment with OH-Cbl 3×2 mg/week IM, betaine 2×6 g PO/day, Folic acid 1×5 mg PO/day was initiated and resulted in resolved cognitive and psychiatric symptoms and improved myelopathy. Biochemical response was marked; tHcy values and MMA excretion immediately decreased while Met remained rather low. Therefore, OH-Cbl treatment was adapted to 3×5 mg IM/ week and methionine 2×125 mg/d was supplemented. Following this scheme, the patient widely recovered but residues of myelopathy remained. His main complaints are impairment of sensory function and hypoaesthesia in the gluteal region and predominantly the lower legs while motor functions recovered almost fully. Evoked potentials show a significant improvement but no resolution of the axonal damage. No thrombosis occurred during four years of follow-up; renal, liver and cardiac function as well as visual acuity and ophthalmoscopy findings were normal. THcy concentrations remain stable between 50 and 60 μmol/L, Met in the upper normal range between 40 and 50 μmol/L and MMA excretion has decreased to 65 mmol/mol creatinine *(reference range <10)*.

#### Case 3 Apathy, reversible white matter abnormalities, paresis and respiratory insufficiency

A 34-year-old woman of Moroccan descent was admitted to the neurology department for evaluation of increasing apathy, confusion and tetraparesis. Family history was unremarkable and the patient did not smoke, drink alcohol, or use illicit drugs.

Weakness and an unsteady, staggering gait together with loss of tendon reflexes of the lower extremities had occurred two years earlier, at that time identified as sensorimotor demyelinating polyneuropathy. Cerebrospinal fluid (CSF) analysis was normal and serologic testing for antiganglioside antibodies was negative. Treated with intravenous immune globulin the patient made a remarkable recovery. In the recovery phase, an anxiety disorder was diagnosed. However, over the next 12 months, the classification of her psychiatric illness was revised several times and various combinations of psychiatric medications were prescribed.

Approximately four weeks before admission, during a stay in Morocco, the patient discontinued her psychiatric medications and was acutely hospitalized due to symptoms consistent with neuroleptic malignant syndrome. In the following, after her return from Morocco her family noticed increasing lethargy and weakness, resulting in inability for self-care and communication.

Based on neurophysiological testing acute axonal polyneuropathy was diagnosed. In addition, MRI of the brain with administration of gadolinium revealed large areas of signal abnormality without enhancement predominantly involving the white matter of both hemispheres, and extending to the midbrain structures. CSF analysis yielded minimal increase of lactate, normal cell counts, protein and glucose. Extensive testing for autoimmune diseases as well as infectious and malignancy-associated aetiologies, ophthalmological investigation and analysis of exposure to heavy metals or organic chemicals revealed normal results. An intermittent episode of abdominal pain prompted suspicion of porphyria and indeed, urine and blood tests revealed mild elevation of porphobilinogen and delta-aminolevulinic acid. A treatment course with hemearginate was instituted presumptively until genetic testing for porphyria proved negative.

Despite symptomatic treatment, the patient’s condition progressively declined over the next four weeks resulting in respiratory failure requiring intubation and mechanical ventilation. Further, bilateral deep venous thrombosis occurred despite consistent administration of prophylactic subcutaneous low-molecular-weight heparin.

The patient was transferred to the neurocritical care unit for further evaluation. Repeat MRI of the neuroaxis showed progression of the signal abnormalities with additional involvement of the spinal cord. A systematic review of previous investigations and clinical presentation prompted metabolic workup which revealed elevated plasma tHcy (53.3 μmol/L), low Met (9.6 μmol/L) and MMA-uria (1168 mmol/mol creatinine) in the presence of normal serum vitamin B12 levels. The cblC defect was proven by enzymatic and complementation studies in cultured fibroblasts and *MMACHC* mutation analysis identified a homozygous mutation (c.347 T > C) which has previously been reported [4,22]. Treatment with intravenous injections of OH-cobalamin (1000 μg) was started, daily for two weeks, then three times a week for the next four weeks, followed by weekly supplementation. After four weeks of treatment, brain and spine MRI showed marked reduction of the white matter abnormalities and tHcy, Met and MMA concentrations had returned to normal. The patient improved steadily and could be weaned off the ventilator. After weaning off centrally acting medications the patient was able to follow commands, and within two months, she was able to walk with assistance. However, persistent neuropsychological deficits including confusion and amnesia required aftercare in a residential-care facility following six month of intensive neurological rehabilitation.

## Discussion

This report summarizes the clinical spectrum of the late-onset cblC defect and adds three new cases. The review of thirty-nine reported cases with disease onset >12 months identifies age-associated patterns and reveals that late-onset cblC disease cannot be considered a homogeneous clinical entity.

A distinct group of young children present with a rather uniform disease with HUS and PAH. The course in these children has mostly been rapidly progressive and outcome was deleterious in four out of six children. HUS was the most frequent symptom in children under six years.

From school age and through adolescence the pattern of the disease changes and becomes dominated by psychiatric symptoms, cognitive decline and ataxia with HUS, neuropathy/myelopathy, macrocephaly and seizures. In adults, thrombosis, neuropathy, myelopathy and dementia are the most prominent features accompanied by glomerular pathologies other than HUS and psychiatric symptoms. Macrocytosis or macrocytic anaemia are not consistently reported. At all ages, the disorder may present with acute or insidious onset, and show sudden deterioration as well as long stable phases.

The pathophysiologic mechanisms behind this severe multisystemic disease have not completely been elucidated at present. However it has been shown that demyelination in the cblC defect (and most probable also in acquired vitamin B12 deficiency) seems associated with low levels of S-adenosylmethionine resulting from the impaired remethylation of Hcy to Met [34]. Met deficiency may also be involved in the impairment of cognitive development by hampering methyl group transfers essential for the synthesis of creatine [35], regulation of gene expression, nucleotide metabolism and many other metabolic pathways [36]. Acute encephalopathic features and long-term neurotoxicity have also been attributed to high MMA levels in the brain especially since the hypothesis of extensively high concentrations of dicarboxylic acids in the brain due to a “trapping” mechanism has been developed [37]. MMA is also known to be involved in chronic renal failure [38] by altering and impairing mitochondrial energy metabolism [36]. Vascular disease and thrombosis have initially been attributed to isolated elevation of tHcy concentrations. However, with the observation of HUS as specific clinical pattern of the cblC defect -which has not been observed in classical homocystinuria, a disease causing high levels of tHcy and Met- it has been suggested that the specific involvement of small vessels causing HUS may result from combined high tHcy and low Met concentrations [36]. It is also of note that the characteristic eye disease frequently encountered in the early-onset cblC defect with retinopathy and loss of visual acuity differs significantly from the eye involvement in classical homocystinuria and may therefore not be explained by isolated high tHcy concentrations.

Due to the wide age distribution at disease onset (1.3-44 years; mean: 15; median 12 years) and the variable clinical phenotypes paediatricians, as well as adult physicians (e.g. neurologists, nephrologists, psychiatrists, immunologists, haematologists and haemostaseologists) are involved in the clinical and diagnostic workup of late-onset cblC patients. Like in other rare IEM, time between onset of disease and diagnosis in most cases is extremely long and a subgroup of reported patients even died without diagnosis [13,20]. Since paediatricians have historically covered the field of IEM, adult physicians sometimes are not too familiar with IEM. However, the late-onset cblC defect presents with many of the classical “textbook pictures” for IEM such as unclear alterations of consciousness and general status, unexplained progressive or intermittent neurological disease, unexplained thromboembolism, weight loss and loss of appetite, or a systemic disease involving more than one organ system. Additionally, history often reveals siblings or other family members having suffered or even died from a similar or “unknown” systemic disease, pointing towards the genetic origin of the disease. Reduction of the diagnostic delay by raising awareness for IEM and lowering the threshold for metabolic investigations would be of significant benefit for the patients, because in contrast to the majority of early-onset cblC patients, most late-onset patients respond to treatment with significant improvement not only of biochemical parameters but also of clinical manifestations. Earlier initiation of treatment could potentially ameliorate the frequently reported neurological sequelae. Therefore, the establishment of adult metabolic clinics and/or close cooperation of metabolic specialists with general paediatricians and adult physicians are a medical need.

In a large series of 88 patients with the cblC defect (including 10 late-onset patients), a male to female ratio of 1.93 was observed. Since the late-onset group in this series encompassed five females and five males, the uneven distribution is determined by the early-onset subgroup [4]. In the reports summarized here, the male to female ratio of 0.7 indicates that in contrast to the early-onset form, the late-onset form of the cblC defect is more frequently reported in females. However, both the series reported by Fischer et al. and the present sample carry high risk of selection and reporting/publication bias. Therefore the observed gender differences need to be confirmed by samples generated e.g. from the recently installed European network and registry for homocystinurias and methylation defects (https://www.ehod-registry.org/). Furthermore, there is no evidence that females in general might have a milder disease course. Reports on two pairs of brothers [10,28] and one pair of sisters [20] clearly demonstrate a significant variation of clinical symptoms between siblings. Furthermore Boxer et al. report the case of a 42 year-old male with late-onset cognitive impairment and his sister who had died from severe, early-onset, progressive neurological disease at age seventeen [30].

The three new exemplary cases illustrate the spectrum of late-onset cblC disease in the adolescent/adult; underline the long delay from first symptoms to diagnosis, and demonstrate both the impressive response to treatment and the sequelae associated with delayed diagnosis in this disorder. This observation suggests that diagnosis by NBS and initiation of early treatment might result in complete prevention of disease symptoms. However, since experience with NBS for cblC [8] is very limited, this question requires future research efforts.

Acute and chronic psychiatric symptoms either isolated or accompanied by involvement of other organs or on basis of impaired neurodevelopment have been described in a large number of IEM in adolescents and adults [39]. Since awareness for rare IEM presenting beyond infancy is still limited also in psychiatric specialists, many patients remain undiagnosed and without specific treatment for years. Generally, indicators for metabolic workup in patients with psychiatric symptoms are fluctuation of symptoms and especially aggravation of disease during catabolism, association with systemic and/or neurological disease and abnormalities (e.g. white matter alterations) revealed by imaging studies of the central nervous system [22,39].

In patients with predominantly neurological manifestations such as myelopathy and neuropathy, clinicians often feel reminded of diseases associated with e.g. alimentary vitamin B12 deficiency, especially when macrocytosis or macrocytic anaemia accompany neurological disease. Nevertheless, as soon as normal cobalamin concentrations in blood are measured, the hypothesis is often dismissed. To avoid this pitfall, the measurement of MMA in urine or in case of impaired renal function also in plasma, completed by measurement of tHcy in blood must be highlighted as the most precise method to detect any functionally relevant “vitamin B12- associated problem” [40,41].

For several years, extensive tHcy measurement had been recommended on basis of an assumed association of mildly elevated tHcy concentrations with major common diseases such as cardiovascular disease, dementia, thrombosis and stroke. Recently, tHcy assessment has been disregarded in many settings due to the failure of large epidemiological studies to prove these associations for the general population [42-44]. Consecutively, even in young, otherwise healthy adults with thrombosis, or in early onset dementia, it might well be that tHcy is not assessed. It must be kept in mind that this approach will lead to a significant number of patients, encompassing not only cblC patients but also individuals with other IEM causing hyperhomocysteinemia (e.g. classical homocystinuria) being missed. One positive exception in this regard is the recent guideline for the workup in patients with atypical HUS, which addresses intracellular disorders of cobalamin metabolism as a differential diagnosis [45].

## Conclusion

The late-onset cblC defect with disease onset beyond the typical infantile presentation and IEM in general are a neglected field of clinical perception and medical workup. Raising awareness for this group of disorders has the potential to improve patients’ outcome and perspective by timely initiation of targeted treatment.

## Abbreviations

Cbl: Cobalamin
cblC: Cobalamin C
HUS: Haemolytic uraemic syndrome
IEM: Inborn error of metabolism
Met: Methionine
MMA: Methylmalonic acid
NBS: Newborn screening
tHcy: Total homocysteine
